# Elements of Dental Materia Medica and Therapeutics

**Published:** 1879-02

**Authors:** 


*Elements of Dental Materia Medica and Therapeutics, with Pharmacopœia.* By James Stocken, L. D. S., R. C. S., England. —Publishers: Lindsay & Blakiston, Philadelphia.

This is a second edition, which is improved by the addition of Tables relating to Weights and Measures, Symbols, Poisons and Notation, increasing the number of pages from 147 to 319 We also note the addition of such agents as black snake root, aloes, carbonate of ammonia, valerianate of ammonia, iodide of ammonia, amylene, amyl hydras, aqua-fortis, armenian bole, sulphate of beberia, calendula, calomel, cannabis indica, animal charcoal, bromine, chlorinated lime, citrate of iron and quinine, colchicum, cold as a remedial agent, corrosive sublimate, cuttle fish bone, digitalis, ether, preparations of iron, golden seal, heat as a remedial agent, preparations of mercury, hypophosphites, iodide of potassium, mechanical remedies, magnetism, pellitory, plaster of paris, acetate of lead, salicylic acid, stramonium, valerian, together with a much better arranged index. These additions add greatly to the value of the work, which is printed in a convenient form and with large type.

